# Nanostructured Lead Sulphide Depositions by AACVD Technique Using Bis(Isobutyldithiophosphinato)Lead(II) Complex as Single Source Precursor and Its Impedance Study

**DOI:** 10.3390/nano10081438

**Published:** 2020-07-23

**Authors:** Sadia Iram, Azhar Mahmood, Effat Sitara, Syeda Aqsa Batool Bukhari, Syeda Arooj Fatima, Rubina Shaheen, Mohammad Azad Malik

**Affiliations:** 1 School of Natural Sciences, National University of Sciences and Technology, Islamabad 44000, Pakistan; sadia.iram@sns.nust.edu.pk (S.I.); effat.sitara@sns.nust.edu.pk (E.S.); aqsa.batool@sns.nust.edu.pk (S.A.B.B.); 2 Department of Materials, University of Manchester, Manchester M13 9PL, UK; azad.malik@manchester.ac.uk; 3 Physics Division, Pakistan Institute of Nuclear Science and Technology (Pinstech), P.O. Nilore, Islamabad 45500, Pakistan; syedaarooj80@yahoo.com (S.A.F.); rubina_shahin_2003@yahoo.com (R.S.)

**Keywords:** lead chalcogenides, aerosol assisted chemical vapor deposition, nanostructures, impedance spectroscopy

## Abstract

This communication reports the synthesis of bis(diisobutyldithiophosphinato)lead(II) complex and its subsequent application as a single source precursor for the nanostructured deposition of lead sulphide semiconductors and its impedance to explore its scope in the field of electronics. Synthesized complex was characterized by microelemental analysis, nuclear magnetic resonance spectroscopy, infrared spectroscopy and thermogravimetric analysis. This complex was decomposed using the aerosol-assisted chemical vapour deposition technique at different temperatures to grow PbS nanostructures on glass substrates. These nanostructures were analyzed by XRD, SEM, TEM and EDX methods. Impedance spectroscopic measurements were performed for PbS in the frequency range of 40 to 6 MHz at room temperature. In a complex impedance plane plot, two relaxation processes were exhibited due to grains and grain boundaries contribution. A high value of dielectric constant was observed at low frequencies, which was explained on the basis of Koops phenomenological model and Maxwell–Wagner type polarization. Frequency-dependent AC conductivity results were compliant with Jonscher power law, while capacitance–voltage loop had a butterfly shape. These impedance spectroscopic results have corroborated the ferroelectric nature of the resultant PbS nanodeposition.

## 1. Introduction

Lead chalcogenides (PbX, X = S, Se, Te) are important semiconductor materials with narrow direct band gaps and some unique properties. For example, over a wide range of nonstoichiometry, their lattice structure remains stable, and they also exhibit positive temperature coefficients (dEg/d*T*) as compared to all other semiconductors, which have negative temperature coefficients. Lead sulfide belongs to the chalcogenides semiconductors family, having a direct bulk band gap of 0.41 eV at room temperature [1]. This narrow band gap widens its application in photovoltaic cells [2], infrared detectors [3], thermoelectrics [4], dielectrics [5], gas sensing [6], biosensing [7] and photocatalytic applications [8]. These attributes are all determined by morphology, surface properties, crystal defects, phase and size, which rely completely on their synthetic methods. PbS nanostructures have been prepared using different methods such as chemical deposition [9], hydrothermal [10], microwave/sonochemical [11], thermal decomposition [12] and sol–gel [13]. Many researchers have investigated the ferroelectric, dielectric and electrical properties of PbS formed by various synthetic techniques. Shanker and coworkers [14] have employed oriented attachment of colloidal ligand-free PbS and studied flexible electronic grade semiconductors. Mild annealing at 150 °C increased the conductivity of PbS nanocrystals. Charge transport properties remained similar after the repeated bending of the PbS on a flexible polymer substrate. A simple hydrothermal synthesis of single-crystalline cubic shaped PbS nanostructures and their photoconductivity and dielectric studies was performed by Sakthivel et al. [15]. This provided evidence of polarization effects and transport properties of the charge carriers. They also observed a blue shift in the band gap compared to the bulk PbS due to exciton confinement. A nanocrystalline deposition of PbS with different nm thicknesses (400, 600) was prepared via the chemical bath deposition technique on glass and Si substrates by Nasir et al. [16]. The conductivity of all PbS depositions was found to be p-type by Hall measurements. It was observed that DC conductivity, capacitance, photocurrent, carrier’s concentration, mobility and drift velocity increased with the increase of thickness.

Toxicity of bisisobutylphosphinato lead(II) complex is not reported in literature due to limited research work investigated so far. Generally, lead sulfur metallo-organic complexes are associated with toxicity concerns. However, Aerosol Assisted Chemical Vapour Deposition (AACVD) evades this concern and circumvents the escape of harmful lead alkyls as compared to conventional chemical vapor deposition which introduces nonstop throw of the precursor’s vapors by using reduced pressure [17]. Lead is a heavy metal, and like all heavy metals, it is more toxic in the metallic form than the lead chalcogenide form. Therefore, the use of lead sulfate is permitted in lead acid charge accumulators [18]. In current work, the deposited PbS nanostructure is highly stable and has less chance of Pb emission; hence, it is safer. As far as PbS toxicity is concerned, it is often described as a benign material and considered to be at the lowest end of toxicity among the binary semiconductor materials. Hence, the use of PbS is far safer than the use of CdS/CdSe or TeS/TeSe.

The current study involves the synthesis of bis(diisobutyldithiophosphinato)lead(II) complex and its employment as a single source precursor for the deposition of PbS nanostructures using the AACVD technique. There is a clear difference in the morphology of the nanostructured deposition compared to those previously reported from dithiocarbamates or xanthates precursors [19,20]. The behavior of each single source precursor is different when used for the deposition of nanostructure by Chemical Vapour Deposition (CVD) because the decomposition temperature of each precursor is different as evidenced by their TGA patterns. This work is the first instance of bis(diisobutyldithiophosphinato)lead(II) complex employment for PbS nanostructure development by AACVD and its ferroelectric, dielectric and electrical parameter studies.

## 2. Materials and Methods

All the materials viz. sodium diisobutyldithiophosphinate, lead(II)acetatetrihydrate salt, toluene, acetone, ethanol and tetrahydrofuran were purchased from Sigma Aldrich, Manchester, UK. All chemicals were of reagent grade and used without further purification.

### 2.1. Synthesis of Bis(Diisobutyldithiophosphinato)lead(II) Complex

Bis(diisobutyldithiophosphinato)lead(II) complex was synthesized by modifying the method described by Kutchen et al. [21]. Diluted aqueous solution of sodium diisobutyldithiophosphinate (NaiBu_2_(PS_2_)) was added drop wise into aqueous solution of lead (II) acetate trihydrate salt and stirred continuously. Resultant precipitates were filtered under a vacuum and dried. Recrystallization via toluene/acetone system furnished bis(diisobutyldithiophosphinato)lead(II) complex crystals. Resultant lead complex showed solubility in most organic solvents including ethanol, toluene and tetrahydrofuran while exhibiting stability in an open atmosphere for a period of months. Elemental percentage calculated for C_16_H_36_PbP_2_S_4_ (*MW* = 625.85) were C (30.71%), H (5.8%), S (20.49%), P (9.9%) and Pb (33.11%) while experimental values were C (30.68%), H (5.4%), S (20.42%), P (9.90%) and Pb (33.10%). IR (cm^−1^) Figure S1: 2916 asym CH_2_ stretch, 2902 sym CH_3_ stretch, 2882 sym CH_2_ stretch, 2074 asym CH_3_ stretch, 1470 CH_2_ bend, 1410 CH_3_ bend, 1300 (P–C), 602 (Pb–S), 760 (P–S). ^1^H-NMR (δ, CDCl_3_, 400 MHz) Figure S2: 1–1.1 (d, 6H; CH_3_), 1.8–1.9 (d, 2H; CH_2_), 2.2–2.4 (m, 1H; CH). (ES-scan) m/z 384 (100%). ^13^C-NMR (CDCl_3_) Figure S3: 24 ppm (CH_3_), 51 ppm (CH_2_), 76 ppm (CH). MP = 180 °C. Yield: 2.90 g, 22.74%.

### 2.2. AACVD Procedure

AACVD apparatus was rigged to perform the nanostructure deposition experiment. THF (10 mL) was poured into a two-neck 100 mL round-bottom flask. Afterwards, 0.20 g (0.8 mmol) bis(diisobutyldithiophosphinato)lead(II) complex was dissolved into it. A carrier gas (argon) inlet with a platon flow regulating gauge was also connected to assist in the transfer of aerosol from the solution flask to the tube reactor. Reinforced tubing was used to join the flask and the reactor tube. The reactor tube was charged by 06 glass slides (approx. 1 × 3 cm) and subsequently placed inside a Carbolite furnace. A round-bottom flask carrying precursor solution was placed in a water bath of piezoelectric modulator of a PIFCO ultrasonic humidifier (Model 1077). Carrier gas swiped droplets of precursor aerosol into the reactor’s hot-wall zone where a nanostructure of PbS was produced by decomposition of bis(isobutyldithiophosphinato) lead complex. Deposition experiments were performed at 400, 450 and 500 °C for 60 min with 200 SCCM argon flow rate under atmospheric pressure. In situ oxidation was inhibited by passing argon through the reactor for 10 min at the temperatures of the deposition experiment. The resultant grey/black deposition of PbS was strongly adhered to the substrate.

### 2.3. Impedance Spectroscopic Analysis

Ideally, a deposition with 1: ≤1 Pb/S stoichiometry i.e., the least Pb rich deposition has good impedance features. Therefore, PbS deposited at 450 °C experiment temperature was selected for impedance spectroscopic analysis because other depositions at 400 °C or 500 °C were highly Pb rich as exhibited by EDX results. Deposited PbS powder was scratched and pressed into a pellet of 12 mm diameter and 1.2 mm thickness. The pellet was sintered in a muffle furnace at 160 °C for 4 h. Electrical contacts were made on both sides of the pellet by using conducting silver paste to form a parallel plate capacitor. Impedance measurements were carried out using an Agilent 4294 LCR Meter (Agilent, CA, USA). Fitting of the measured result was performed using ZView software (North Carolina, 3.2 Version).

### 2.4. Structural and Microstructural Characterization

FTIR analysis was performed using a Bruker platinum ATR model Alpha Germany (Bruker, Karlsruhe, Germany) within a spectral range of 550 to 4000 cm^−1^. Thermogravimetric analysis and elemental analysis was carried out at the Microanalytical Laboratory of University of Manchester, using a Thermo Scientific Flash 2000 (Thermo fisher scientific, Waltham, MA, USA) organic elemental analyzer and a Seiko SSC/S200 (Seiko, Ginza, Japan) under Nitrogen gas. TGA was attained from room temperature to 600 °C at a heating rate of 10 °C min^−1^. A Bruker D8 Advance diffractometer (Bruker, Karlsruhe, Germany) equipped with a Cu-Kα source was used to get X-ray diffraction patterns. ^1^H-NMR was carried out at a Bruker AVANCE III 400 MHz spectrometer (Bruker, Billerica, MA, USA). ^13^C-NMR was acquired by a Bruker AVANCE 300 MHz spectrometer (Bruker, Billerica, MA, USA). Scanning electron microscopy (SEM) was done by an FEI XL-30 scanning electron microscope (FEI, Hillsboro, OR, USA). Transmission electron microscopic analysis was carried out via a Tecnai F30 FEG TEM instrument (FEI, Hillsboro, OR, USA). Impedance spectroscopic studies were performed by an Agilent 4292 LCR METER (Agilent, CA, USA).

## 3. Results

### 3.1. Spectroscopic and Gravimetric Analysis

**Figure 1:**
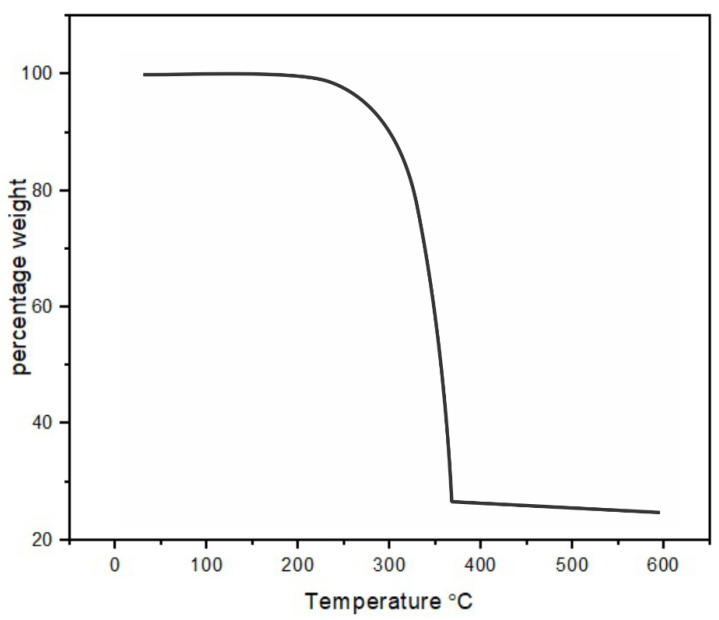
Thermogravimetric analysis graph of [Pb(iBu_2_PS_2_)_2_].

**Figure 2:**
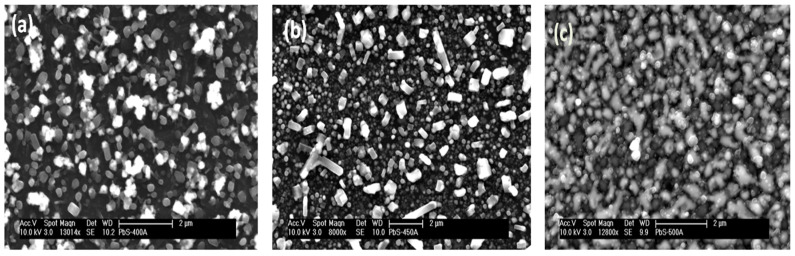
SEM images of PbS thin depositions at: (**a**) 400 °C; (**b**) 450 °C; (**c**) 500 °C.

TGA analysis of [Pb(iBu_2_PS_2_)_2_] complex has furnished mass losses in multiple steps between 220 and 380 °C at 10 °C.min^−1^ under N_2_ atmosphere (Figure 1). Decomposition onset temperature was observed near 220 °C along with sublimation while decomposition was completed at about 380°C. SEM images of deposited PbS formed at 400, 450 and 500 °C from the [Pb(iBu_2_PS_2_)_2_] precursor are shown in Figure 2. The PbS deposited at 400 °C showed cubical morphology on the whole surface of the glass substrate. Comparable morphologies were found at 450 and 500 °C; however, the depositions were denser at higher temperatures. SEM micrographs depicted cubical morphology in a uniform fashion corroborating the formation of PbS cubes at all temperature conditions of the experiment. These depositions were too thin despite increasing the quantity of the precursor, mainly due to the lower solubility of bisisobutylphosphinato lead complex in the solvent system as compared to the dithiocarbamato or xanthate complexes. EDX analysis has shown these depositions were composed of lead and sulfur in ratios of 89:11, 64.7:35.3 and 87:13 at 400, 450 and 500 °C reactor temperatures, respectively. These nonstoichiometric ratios remained almost the same in three repeated trials by EDX. This finding coincides with the work of O’Brien et al. [22], who reported synthesis of bis(diisobutyldithiophosphinato)cadmium(II) precursor to grow thin depositions of CdS. The energy dispersive analytical X-ray (EDAX) pattern gave peaks for cadmium and sulfur that indicated a slight excess of Cd (1.00:0.97). This may be due to a different vaporization rate owing to the significant difference in boiling points of heavy metals and sulfur.

**Figure 3:**
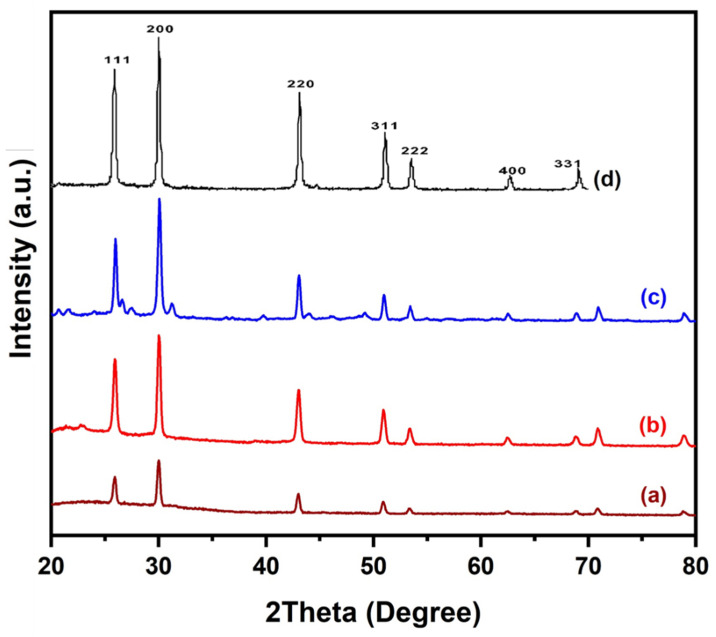
XRD pattern of as-deposited PbS at: (**a**) 400 °C; (**b**) 450 °C; (**c**) 500 °C; (**d**) Reference pattern of PbS [23,24].

Figure 3 shows an x-ray diffraction (XRD) spectrum of deposited PbS with space group Fm-3m (225). XRD spectrum was achieved using Cu-Kα x-rays of voltage 40 Kv and current intensity of 30 mA. All diffraction peaks in Figure 3 were indexed to a pure cubic phase of PbS JCPD No. 03-065-0692. Small peaks of glass substrate were also exhibited in an XRD spectrum of PbS 500 °C due to incomplete coverage, indicating that the deposition was very thin. No extra peaks of impurities were detected in these spectra.

**Figure 4:**
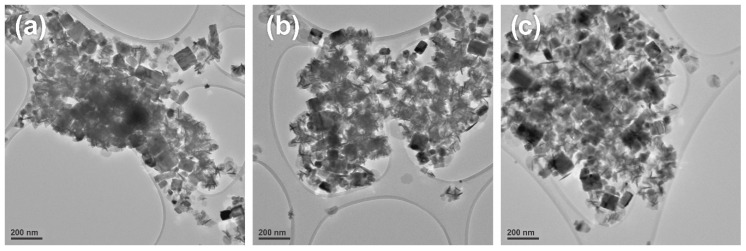
TEM images of as-deposited PbS at: (**a**) 400 °C; (**b**) 450 °C; (**c**) 500 °C.

TEM images of as-deposited PbS from precursors at different temperatures are shown in Figure 4a–c. It was also clear from the TEM graphs that nanoparticles contain cubical morphology and corroborate the findings of XRD and SEM analysis. These deposited nanoparticles were distinct and uniform in size: less than 200 nm i.e., in nanometric range, as shown by TEM images.

### 3.2. Impedance Analysis

Impedance spectroscopy is a powerful technique to study detailed electrical conduction mechanisms and the dielectric properties of the materials. Impedance analysis was performed at room temperature in the frequency range of 40 Hz to 6 MHz. The real part of impedance *Z*′ and the imaginary component *Z*″ are represented as

*Z*’ = *R*/1 + (ω*RC*)^2^ (1)

*Z*’’ = *R*(ω*RC*)/1 + (ω*RC*)^2^ (2)

where *R*, *C* and ω are resistance, capacitance and angular frequency, respectively.

**Figure 5:**
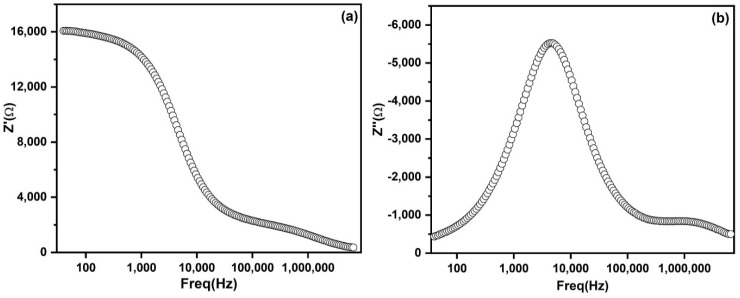
Impedance plot of PbS: (**a**) Variation of *Z*′ vs. frequency; (**b**) *Z*″ vs. frequency at room temperature.

Figure 5a,b demonstrates the variation of real part (*Z*′) and imaginary part (*Z*″) of impedance as a function of frequency. In the *Z*′ vs. frequency plot, a frequency independent region was observed in the low frequency region, which signifies the DC invariant electrical conductivity [25]. *Z*′ exhibited a higher value in low frequency and subsequently decreased monotonically with frequency to attain a nearly constant value at higher frequencies. This can be attributed to the release of space charge polarization [26].

**Figure 6:**
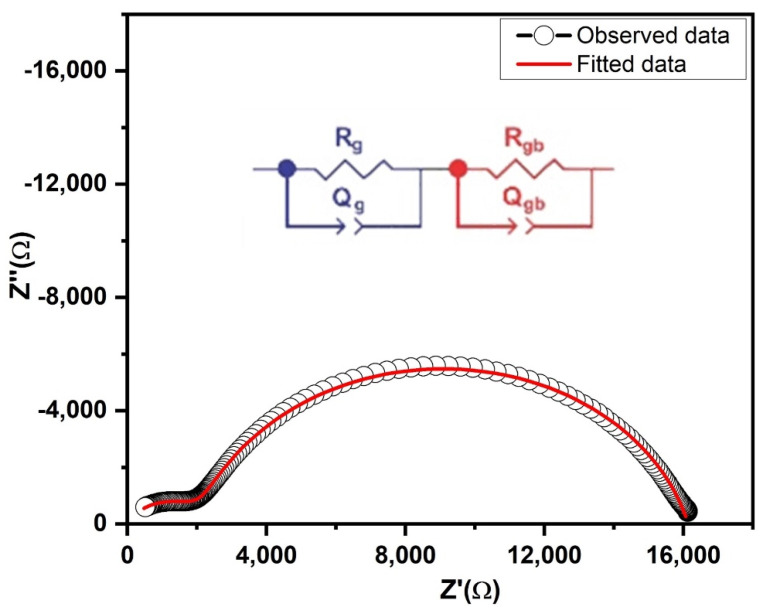
Complex impedance plane plot of PbS and Resistor-Capacitor circuit used for fitting.

Since *Z*″ = *CR*^2^, the imaginary part of impedance as a function of frequency magnifies the most resistive element of the circuit. Two peaks were clearly observed in the plot which identified the presence of at least two different relaxation processes. The Nyquist plot (*Z*′ vs. *Z*″) was used to study the individual contributions from grains, grain boundaries and electrode contact to electrical properties of the sample. Each semicircular arc in the Nyquist plot represented a unique relaxation process. Two relaxation processes with quite different relaxation times (τ = *RC*) were evident from the appearance of two well resolved semicircles (Figure 6). Higher and lower frequency semicircles corresponded to grain conduction and grain boundary conduction, respectively. Both semicircles were depressed with their centers lying below the real axis of impedance which is indicative of non-Debye type dielectric behavior [27]. An equivalent circuit model (R_g_Q_g_)(R_gb_Q_gb_) was employed to fit the impedance data by ZView software. Constant phase element (CPE) was used to address the non-ideal behavior of the capacitor. The capacitance of these electroactive regions was calculated by *C* = *R*^(1−n/n)^*Q*^1/n^ where n is zero for pure resistor and one for pure capacitive behavior [28]. The fitted results are presented in Table 1.

The value of AC conductivity is in the range of 10^−3^ S/m which is the same as reported earlier for the PbS nanosheet prepared by solid state method [5]. However, it is greater than PbS nanomaterial synthesized by hydrothermal method, which is in the range of 10^−7^ S/m [29].

**Figure 7:**
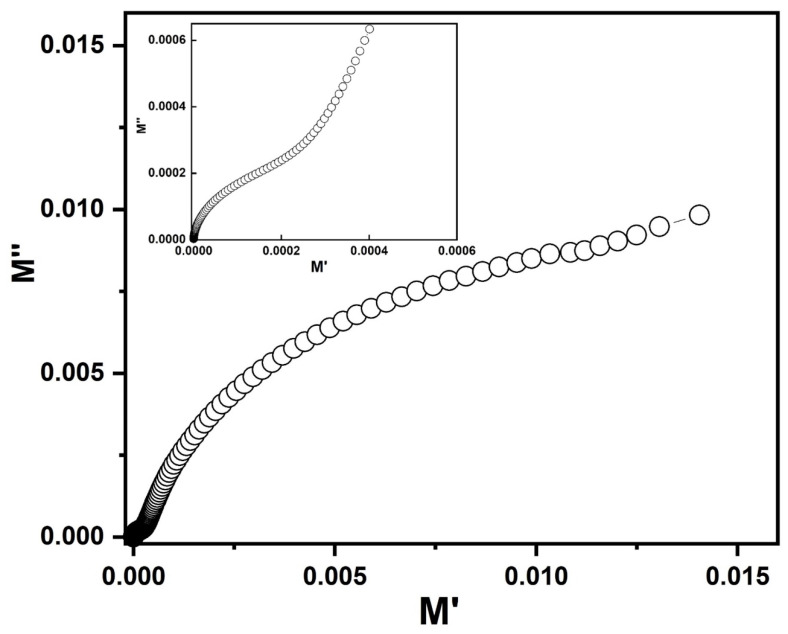
Complex modulus plane plot (*M*″ vs. *M*′) at room temperature for PbS.

The complex modulus (*M*′ vs. *M*″) formalism approach was employed to study the electrical properties of the materials. The real and imaginary parts of the modulus were represented as *M*’ = ω*C*_0_*Z*’’ and *M*’’ = ω*C*_0_*Z*’, where *C*_0_ = ε_0_*A*/*t* (*A* is the area and *t* is the thickness of the sample) [30]. The electric modulus suppressed the extrinsic effects such as electrode-polarization and magnified the bulk behavior [31]. From the *M*′ vs. *M*″ plot two semicircles were observed as depicted in Figure 7. A larger semicircle in the high frequency field was attributed to the grain effect while a low frequency semicircle signified the grain boundary effect.

**Figure 8:**
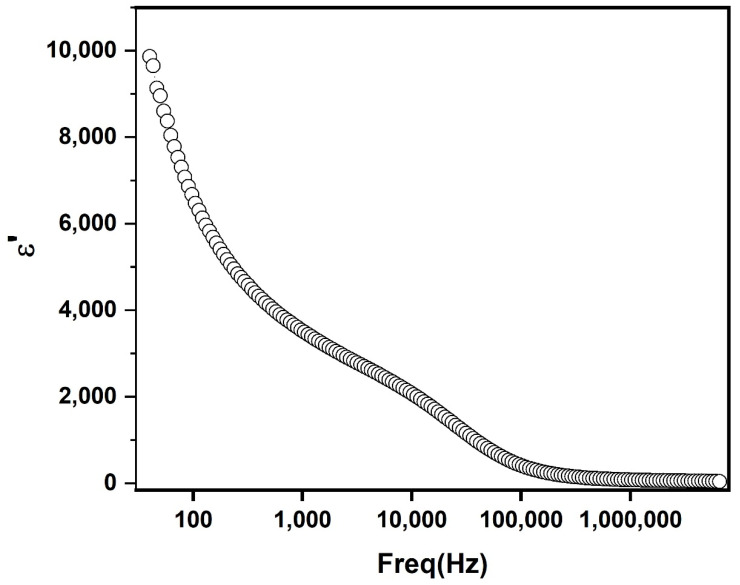
Variation of real part of dielectric constant with frequency for PbS.

Variation in the real part of the dielectric constant with frequency indicated the presence of more than one type of polarization process as illustrated in Figure 8. A higher value of dielectric constant was observed in the lower frequency region, which subsequently decreased with high frequency because dipoles at higher frequencies cannot follow the field and thus resulted in a decrease of dielectric constant. This became frequency independent above 10^5^ Hz due to a bulk effect. Higher values of dielectric constant at lower frequencies can be explained on the basis of Koops phenomenological model and Maxwell–Wagner type polarization as dielectric materials are supposed to consist of poor grain boundaries that contribute more at low frequencies and are separated by good conducting grains [32]. Dispersion observed at lower frequencies, i.e., below 10^2^ Hz was attributed to interfacial polarization while that above 10^4^ Hz was due to dipolar polarization. At higher frequencies, i.e., above 10^5^ Hz, electronic and atomic polarizations contributed to dielectric constant [33].

**Figure 9:**
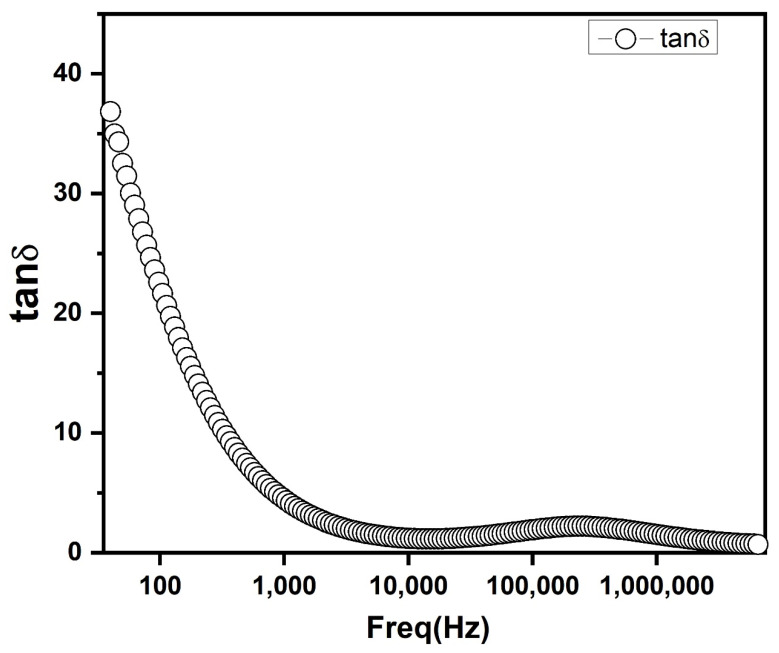
Tan loss as a function of frequency for PbS.

A broad relaxation peak was observed in dielectric loss (tan δ) vs. frequency plot (Figure 9). This is due to energy loss by conduction within the material. Tan loss arises when polarization lags behind the applied alternating electric field [31].

In order to study the electrical transport mechanism, AC conductivity was calculated from the measured impedance data by using following relation

**Figure 10:**
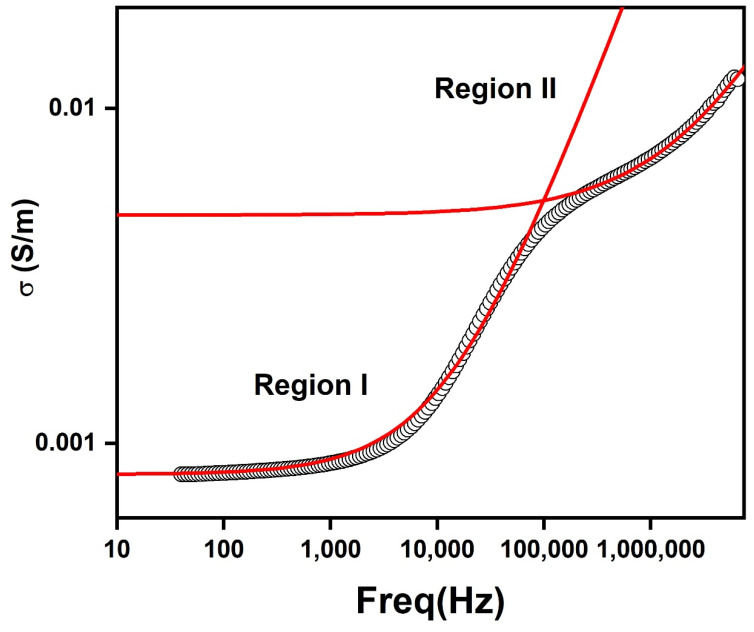
Power law fitting of frequency dependence of AC conductivity for PbS.

σ’ = [*Z*’^2^/(*Z*’^2^ + *Z*’’^2^)] × (*t*/*A*) (3)

where ‘*t*’ is the thickness and ‘*A*’ is the area of the sample. Two frequency independent regions were observed in AC conductivity vs. frequency plot as presented in Figure 10. These low and high frequency plateaus signify the contribution of DC conductivities of grain boundaries and grain respectively. At a certain hopping frequency, the conductivity began to increase with frequency. The AC conductivity curve was fitted by Joncher’s power law for two regions

σ_(ω)_ = σ_o_ + *A*ω*^n^* (4)

where σ_o_ represents the frequency independent DC conductivity and the term *A*ω*^n^* corresponds to frequency dependent AC conductivity. ‘*A*’ is the pre-exponential factor and *n* is the frequency exponent which describes the degree of interaction of mobile charges with the lattice. Values of ‘*n*’ less than 1 corresponded to translational motion of charge carriers, while ‘*n*’ values greater than one indicated the localized hopping mechanism [34]. Values of n_1_ and n_2_ for regions I and region II were 0.85 and 0.65, respectively. The fitted results are shown in Table 1.

**Figure 11:**
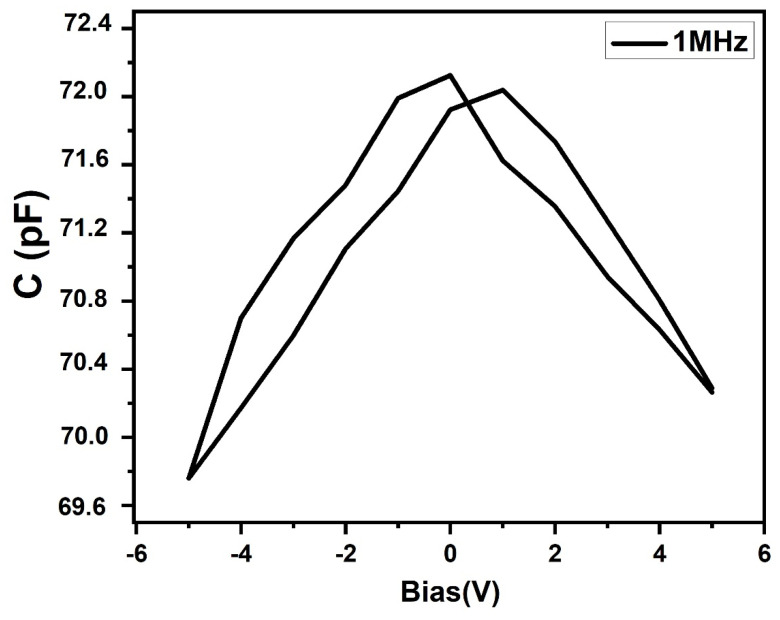
Capacitance–Voltage plot of as-deposited PbS at 1 MHz.

To investigate the ferroelectric behavior of the sample, capacitance–voltage measurements were carried out at room temperature by varying the DC electric field from +5 V to −5 V at 1MHz applied frequency. A characteristic butterfly shaped Capacitance–Voltage loop (Figure 11) was observed which indicated a ferroelectric polarization switching [35].

## 4. Conclusions

Bis(diisobutyldithiophosphinato)lead [Pb(iBu_2_PS_2_)_2_] complex was successfully synthesized and characterized by IR, elemental analysis, ^1^H and ^13^C-NMR. Thermogravimetric analysis profiles for [Pb(iBu_2_PS_2_)_2_] showed decomposition onset temperature near 220 °C where it started sublimation and completed decomposition near 380 °C. The AACVD method was used to develop nanostructures of PbS from [Pb(iBu_2_PS_2_)_2_] at 400–500 °C. The XRD, SEM and TEM results corroborated that cubic crystal structures of PbS were produced at all temperature ranges of the experiment.

Impedance spectroscopic analysis showed two well resolved but depressed semicircles in the nyquist plot (*Z*’ vs. *Z*’’) which indicated the non-Debye type dielectric behavior of the sample. An equivalent circuit model (R_g_Q_g_)(R_gb_Q_gb_) was used to fit the impedance data. The values obtained for R_g_ and R_gb_ were 2.1 × 10^3^ Ω and 14.0 × 10^3^ Ω, respectively. In the low frequency region, high values of dielectric were observed which decreased with increasing frequency and became frequency independent above 10^5^ Hz, corresponding to bulk effect. The variation of AC conductivity with frequency presented two plateaus. The frequency-dependent AC conductivity was fitted by Jonscher’s power law to extract the values of *n*_1_, *n*_2_, σ_gb_ and σ_g_. A butterfly shaped C–V loop suggested the ferroelectric property of the PbS sample. These impedance results and the ferroelectric nature of the resultant PbS nanocrystal product make it a potent candidate for many technological applications like manufacturing of capacitors, wave guides, storage memories and bio and chemical sensors.

## Figures and Tables

**Table 1:** Impedance and AC conductivity fitting results of PbS.

Impedance Fitting Results								AC conductivity Fitting Results			
*R_g_* (Ω)	*Cg* (F)	*R_gb_* (Ω)	*C_gb_* (F)	*n_g_*	*n_gb_*	τ*_g_* (s)	τ*_gb_* (s)	σ*_gb_* (S/m)	σ*_g_* (S/m)	*n*_1_	*n*_2_
2118	4.94 × 10^−11^	14,023	2.59 × 10^−9^	0.72	0.84	1.04 × 10^−7^	3.63 × 10^−5^	8.08 × 10^−4^	4.8 × 10^−3^	0.85	0.65

## Supplementary Materials

The following are available online at https://www.mdpi.com/2079-4991/10/8/1438/s1, Figure S1: FTIR of bis(diisobutyldithiophosphinato)lead, Figure S2: ^1^H-NMR of bis(diisobutyldithiophosphinato)lead, Figure S3: ^13^C-NMR of bis(diisobutyldithiophosphinato)lead.
