# Supplementary figures and images for: Nanostructured Lead Sulphide Depositions by AACVD Technique Using Bis(Isobutyldithiophosphinato)Lead(II) Complex as Single Source Precursor and Its Impedance Study
Source: Nanomaterials (Basel). 2020 Jul 23;10(8):1438. doi: 10.3390/nano10081438 (PMC7466212; doi:10.3390/nano10081438)

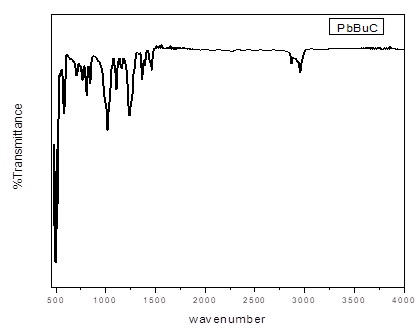

Supplement: Supplementary file 1 [file nanomaterials-10-01438-s001.zip › S1.jpg]

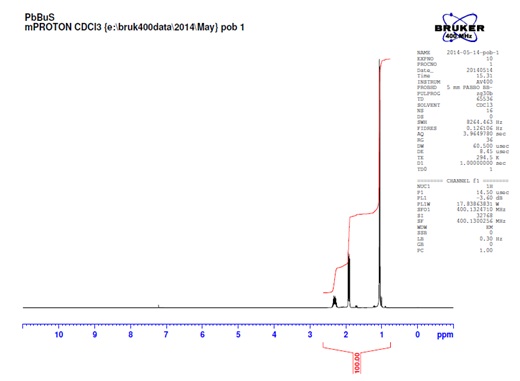

Supplement: Supplementary file 1 [file nanomaterials-10-01438-s001.zip › S2.jpg]

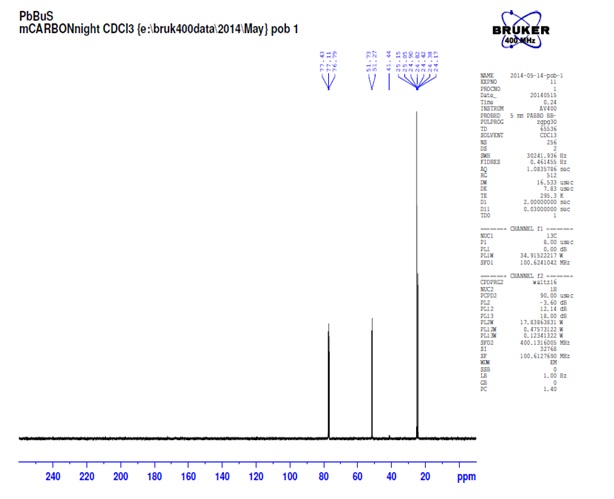

Supplement: Supplementary file 1 [file nanomaterials-10-01438-s001.zip › S3.jpg]
